# Nursing's Relationship to Feminism: What Is Nursing's Response to the Global Backlash Against Women's Rights and Access to Abortion?

**DOI:** 10.1111/jan.70439

**Published:** 2025-12-22

**Authors:** Helen T. Allan

**Affiliations:** ^1^ Emerita Professor of Nursing, Faculty of Health, Social Care and Education Middlesex University London Hendon UK

## 1 Introduction

In this paper, I explore the ambivalent relationship between second‐wave feminism and nursing in the context of attacks on and withdrawal of women's rights and access to abortion (LeBold and MacDonnell 2020). The literature discussed here shows how relevant and ethically suitable feminism is for nursing practice in women's health and abortion services. If there seems to be no one overall response from nursing to abortion, there are nevertheless strong, coherent, ethical and reasoned responses to the ‘global backlash against access to abortion’ (Hayes 2025) based on feminism, which should be more widely known in professional preparation programmes as well as in post‐registration training.

## 2 Women's Rights and Access to Abortion

Access to reproductive health services, including abortion, improves societal health and the social and economic well‐being of women and all people with a uterus (United Nations [U.N] 2014, 2020; Carson et al. 2022). Worldwide, 73 million induced abortions take place annually, with 29% of all pregnancies ending in induced abortion (World Health Organisation [WHO] 2024). While abortion laws vary among countries and over time, the easing of restrictions on abortion is seen as part of a broad social movement towards expanded rights for women (Berer 2017), albeit it remains a stigmatising experience (Maxwell et al. 2021). During the second half of the 20th century, abortion was made permissible within a legal framework that allowed abortion on specified grounds in 100 countries (https://www.globalcitizensolutions.com/the‐abortion‐laws‐which‐countries‐allow‐abortion/). The global North, central and eastern Asia have the most liberal laws on abortion, while Africa, Latin America, the Middle East, and southern Asia (Centre for Reproductive Rights 2024a) have more restrictive laws. Reasons for permitted abortion include the threat of life to the mother, to the child or socio‐economic reasons.

However, despite overall expansion on the legal grounds for abortion, policies remain restrictive in many countries (United Nations 2014). Since 2016, previously established rights and access to abortion have been withdrawn in many countries (Brysk 2025; LeBold and MacDonnell 2020).

In the United Kingdom [U.K.], there is different abortion legislation covering Northern Ireland (Abortion [Northern Ireland] [No. 2] Regulations 2020); Scotland (Abortion [Scotland] Amendment Regulations 2021); and England and Wales. Prior to June 2025, the procurement of an abortion was a criminal offence in England and Wales under the Offences Against the Person Act 1861, although the Abortion Act 1967 provided a legal defence for both the pregnant woman and her doctor where there was:

- Risk to the life of the pregnant woman;
- Risk of grave permanent injury to her physical or mental health;
- Risk of injury to the physical or mental health of the pregnant woman or any existing children of her family (up to a term limit of 24 weeks of gestation); or
- Substantial risk that, if the child were born, they would “suffer from such physical or mental abnormalities as to be seriously handicapped” (United Kingdom Government 2025).

The approved amendment to the British government's crime and policing bill passed by the British Houses of Parliament on Tuesday 17th June 2025 will prevent women from being investigated, arrested, prosecuted or imprisoned for terminating their own pregnancies without changing the existing abortion legislation in England and Wales (Editorial, 15 May 2025, Guardian; Al‐Othman 2025a, Guardian, 2). A rival amendment which sought to legalise abortion was not voted upon (https://voxpoliticalonline.com/2025/06/18/the‐new‐abortion‐law‐what‐it‐means‐why‐it‐matters‐and‐why‐some‐missed‐the‐point/). These amendments follow the unsuccessful prosecution of Nicola Packer who was accused of “unlawfully administering to herself a poison or other noxious thing with the intent to procure a miscarriage” (https://www.bbc.co.uk/news/articles/c93y5gq09x7o); Al‐Othman 2025a.

Under the exemptions above, prosecutions by the police were rare prior to the COVID‐19 pandemic, when online medication for early abortion was permitted under emergency legislation in the U.K. (https://lordslibrary.parliament.uk/coronavirus‐and‐abortion‐law/). However, since the pandemic and the availability of online abortion medication in first‐trimester pregnancy, 100 women have been criminally investigated, six have faced court and one has been sent to prison on suspicion of illegal abortion offences.

It is increasingly the case that in England and Wales, women who suffer a pregnancy loss are treated as suspects and subjected to invasive investigations that inflict profound long‐term harm. One of those prosecuted in 2025 was Nicola Packer, and her case makes salutary reading for nurses and midwives caring for women in similar situations. Packer was criminally prosecuted for illegally terminating her pregnancy in 2021. The case was heard in court in May 2025, and she was found innocent of these criminal charges. She described being arrested by police, ‘I was still in pain [from the abortion], I was extremely tired, I felt very weak and mentally, I just did not understand what was going on’ (Al‐Othman 2025b, Guardian 19). Under guidance from the National Police Chiefs’ Council in 2025, officers were told that they can seize women's phones and search their messages, internet history and even health apps if they are suspected of ending a pregnancy outside of the law (Davis, 18 May 2025, The Observer p5). Packer describes not telling medical or nursing staff initially that she had taken abortion pills ‘because she was afraid it would affect the quality of care she received’. As she says, ‘They didn’t really help me, did they? I think I was right to be frightened’.

Abortion has been politicised in battles over women's reproductive and sexual rights since first‐wave feminism in the late 19th and early 20th centuries (Purcell 2015) see Table 1. The rolling back of legislation and permissions is described as ‘unprecedented threats’ to established women's rights (Feeley et al. 2020). Four governments have rolled back abortion legislation, in some instances banning it altogether: the United States, El Salvador, Nicaragua and Poland (Centre for Reproductive Rights 2024b). The ‘global back lash against abortion’ (Brysk 2025; Finer and Fine 2013; Hayes 2025) has seen an extraordinary withdrawal of women's rights and access to abortion (and other legal, reproductive and social rights) as ‘contradictory trends of 21st‐century abortion rights are driven by disparate nationalist responses to globalisation*’* (Brysk 2025).

**TABLE 1:** Waves of feminist thought 1800s–present day.

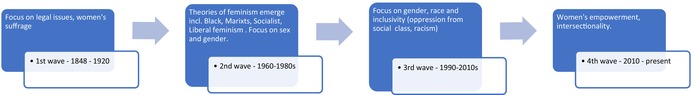

## 3 Feminism and Nursing

It has been observed that the sociological literature is richer than the nursing literature, which is concerned with practice rather than articulating any feminist nursing theory (Allan 1993, 1457). Chinn and Wheeler (1988) between nursing and second‐wave feminism (Malka 2007; Sullivan 2002). As a result of the literature search and review undertaken for this paper, I argue that this relationship remains ambivalent even though the scholarship on feminist theory applied to nursing is far richer than it was in 1993.

There are broadly four waves of feminist theory and action, which I will refer to in this paper (see Table 1 below).

Feminism is not a homogenous theory or movement as the theories and political actions which emerged in second‐wave feminism attest; different emphases continue to be placed by different feminists on the intersecting causes of women's oppression (Melchior 2004) and on social justice and equity (Melchior 2004). While a liberal feminist approach might argue for equality through a level playing field in education and social institutions (Melchior 2004), Marxist feminists would argue for the abolition of capitalism and private property, which they see as the root causes of inequality, including inequality between genders (Mitchell 1971). Socialist feminists would argue that the intersection of capitalism and patriarchy leads to women's oppression and that equality cannot be achieved until the end of economic and cultural oppression, which are overlapping structures that produce an individual's experience of oppression (Gordon 2016).

There continues to be disagreement over feminism and what the future of feminism looks like (Hooks 2015). Yet each tradition within feminism builds on a philosophy and ethics of equality and care which share three broad tenets:

- That men have dominated society cross culturally and historically; this oppression, which is expressed through structural patriarchy, has discriminatory effects on the political, the socio‐economic, the public and the private lives of women and men. Patriarchy oppresses women through the social organisation of reproduction and the sexual division of labour (Weedon 1997). Only recently, have feminists extended this definition of oppression to include women of colour and other marginalised groups (Van Wijlen and Aston 2019).
- That patriarchy and the resulting oppression of women is grounded in essentialist, reductionistic views on nature and biology as fixed (or ‘destined’) (Aranda 2018) which are embedded in and reproduced through biomedical science, ‘the female body as the vessel that mediates male dominance*’* (Shai et al. 2021).
- That patriarchy can only be overcome through a consciousness or an awareness of oppression expressed through valuing the feminine in feminist politics and theory. ‘a movement to end sexism, sexist exploitation and oppression’ (Hooks 2015, 1).

iv Black and Brown women face multiple discrimination as non‐white ethnic women (Nobel 2016).

In its third wave (Melchior 2004), feminism recognises the diversity of experience of women's lives, including the role that racism has played in Black and Brown women's inequality (Brathwaite 2018) long articulated and described by Black feminists yet ignored by white feminists. This theoretical analysis of race, women and patriarchy was developed further in the 2010s during the fourth wave following the emergence of intersectionality theory (Crenshaw 1989, 1991; Iheduru‐Anderson et al. 2021; Siira et al. 2023). Intersectionality has particular resonance for nursing as Brathwaite 2018, a Black British nurse academic, argues:

[The] ‘centrality of whiteness at the expense of the equitable treatment of Black British people continues in all aspects of British healthcare for those employed and those using the service’ (Brathwaite 2018, 920).

Therefore, for the purposes of this paper, I would add to the three tenets above (which derive from Allan 1993 and subsequent thought and work Allan 2021) a further tenet of feminism:

First‐wave feminism in the 19th and early 20th centuries, which emerged globally in writings and actions among women of all social classes and ethnicities, coalesced around a number of health and social justice issues that resonate with nursing practice and scholarship today: the enfranchisement and property rights of women; abolition (of slavery); rejection of gender roles; a focus on women's health, particularly public health, poverty, poor wages and housing, the temperance movement, birth control, sexual health and safer childbearing (Malka 2007; Martz 2020; Fowler 2016; Martz 2020). In the 1960s, second‐wave feminists critiqued the continuing and entrenched sex and gender roles caused by historic as well as new forms of patriarchy and their effects on women's health and social, economic and political position in society (Malka 2007).

Of particular relevance to nursing, a women's health movement emerged in the 1970s as part of second‐wave feminism (Nichols 2000; Roberts et al. 2016). This sought to increase awareness of women's health issues through knowledge of their own bodies outside patriarchal medical knowledge and professional structures such as medicine, nursing and midwifery:

if we don’t learn about our own bodies, somebody else is going to be in charge of what happens to us. It's just that simple (Downer 2011).

Two strands emerged in the women's health movement; the first was active outside healthcare and without healthcare professionals (Paynter et al. 2019; Wild 2024). The second was active inside healthcare (by patients and professionals) in the form of action to recognise and expose sexism, misogyny and patriarchal practices. A tension or ‘entanglement’ (Melchior 2004) at the core of the relationship between second‐wave feminism and nursing is the perceived requirement for nurses to work with doctors and thus their perceived failure to critique and reject patriarchal medical structures, systems and professions (Oakley 1993; Turner 1995; Wall 2007). Some nurses and midwives working within healthcare did critique patriarchy and sought to restructure nursing and midwifery practice (Medhurst 2023) in the 1970s–1990s. Nurse‐ and midwife‐led practice which emerged from the 1990s onwards, for example, birth centres, nurse prescribing, nurse‐led care and advanced practitioners, were all challenges from within nursing and midwifery to medical and patriarchal dominance (Feeley et al. 2020; Malka 2007). Yet the problematic relationship with medicine as a patriarchal structure is reproduced across generations (Malka 2007).

During second‐wave feminism, equality feminism, in the 1960s–1970s, nursing was seen to embody an archetypal gendered profession that reinforced gendered feminine stereotypes (Ashley 1976, cited by Malka 2007, 98). As more women entered the workforce in the 1970s, other occupations offered less gendered choices for young women (Wright‐Mendoza 2018). The gendered image of nursing remains deeply entrenched (Baduge et al. 2024).

In the 1980s, in a later phase of second‐wave feminism, difference feminism, nurse academics and teachers applied feminist theories, typically Gilligan's ethics of care (1982, 1993) to nursing theories on care and caring (Harbison 1992; Malka 2007; Peter and Liaschenko 2020). While feminist‐influenced theories of nursing were embedded in nurse education from the 1980s to the present day (Falk‐Rafael et al. 2004; Michela 2014), some have argued that the discourses and practices of passivity and powerlessness remain embedded both in education and practice to a large extent (Bergh et al. 2015; Paynter et al. 2021; Traynor 2017; Welch 2011). In relation specifically to abortion, LeBold and MacDonnell's 2020 argument is that nursing discourses uphold state‐sanctioned gendered language around abortion which in turn uphold patriarchal state interests:

While nurses have resisted, they have often upheld gatekeeping and decision‐making power reinforcing middle‐class values, often undermining people's agency and knowledge (LeBold and MacDonnell 2020, 83)

Although the provision of abortion medication could be seen as a neoliberal turn (LeBold and MacDonnell 2020) this policy change has done little to loosen reproductive control by states.

LeBold and MacDonnell (2020, 78) describe the nursing literature as relatively silent on abortion and that nurses’ roles in assisting in abortion services are frequently invisible. Nurses’ primary role in abortion services appears to be to provide technical and emotional support to doctors and women (Mainey et al. 2020). In many countries, nurses and midwives prescribe and administer first‐trimester abortion medication (Battistelli et al. 2018; Mainey et al. 2020). And there is evidence that local practice in countries which do not allow nurses or midwives this autonomous practice can be more nuanced, and nurses and midwives may act more autonomously than national legal frameworks would suggest (Mainey et al. 2020; Sheldon and Fletcher 2017):

Some countries interpreted laws ambiguously (Mainey et al. 2020, 1516).

Research in the U.K. conducted recently suggests that abortion by nurse practitioners would be acceptable to British women (Wellings et al. 2024). The authors conclude that the law in the U.K which requires abortions to be authorised and performed by doctors impedes best practice.

Carson et al. 2023 argue that the aim of any feminist abortion education and care is to produce ‘Nurses who are willing to provide abortion care and lead advocacy efforts for equitable, accessible reproductive services face barriers to working to full scope*’* (Carson et al. 2023, 58). However, nurses who work closely allied to and controlled by medicine within patriarchal healthcare systems, may be reluctant to challenge gatekeeping practices and discourses which frame abortion through the lens of patriarchal and professional interests, especially if they are lone practitioners (LeBold and MacDonnell 2020). This reluctance to challenge arises not only because nurses lack power in healthcare systems (Traynor 2017), but also because abortion is framed as a medical problem which empowers doctors as decision makers around access to abortion. LeBold and MacDonnell 2020 argue that framing abortion as a ‘*medical necessity’* reinforces its essentially unacceptable nature in pronatalist and patriarchal societies rather than a social good. In the portrayal of abortion as a medical necessity, representations of alternative values related to abortion (e.g., viewing it as a human right, an economic or social good, a determinant of women's well‐being) are absent from public discourse and even nursing and medical curricula (Carson 2018). Both Paynter et al. 2019 and Carson et al. 2022 point out that even where abortion is legal, positive representations of abortion in education, policy and healthcare systems are missing.

Thankfully, there is evidence of nurses and midwives challenging patriarchy in many areas of women's healthcare where nurses and midwives have access to training and support from within the care context in women's health (Paynter et al. 2019). For example, where the practice milieu is supportive of innovative practice, they are able and willing to challenge patriarchal hierarchies and relationships with doctors (Allan and Barber 2005). Personal choice and reasoning are known to be part of why individual nurses take on new roles. Allan and Barber 2005 found that nurses taking on sperm aspiration in an infertility clinic searched for a moral story that validated their decisions to expand their practice, often in the face of resistance from doctors. (Carson et al. 2022) study showed that nurses challenged doctors’ control over women's reproductive care in relation to the introduction of medication abortion during the COVID‐19 pandemic. In this case, the authors found that a health equity model provided justification for nurses in taking on new roles in abortion.

Although feminist theories were used to underpin some nursing and midwifery curricula in the 1990s–2000s (Diekelmann 2001; Ironside 2001, 2005; Welch 2011), Paynter et al. 2019 found an absence of feminist discourse around abortion in nursing education and training. She cites Haney 2011 to argue that a failure to educate students about nursing roles in abortion services is based on the invisibility of nursing roles in a stigmatised service, on the lingering effects of religious beliefs which underpin the development of both nursing identity and professionalisation. But perhaps more importantly, a failure to educate nurses about abortion may be due to an orientation in nurse education and practice to doing and experiencing, Wall (2007) states that nurses’ exposure to social theory of any type can be limited during nursing education, they and their teachers are ‘likely to shy away from theory that is political’ (p. 42). Given Wall's argument, it is noteworthy that this hasn’t always been the case. In the U.K., the Royal College of Nursing (RCN) and the Royal Colleges of Midwives (RCM) successfully lobbied for the inclusion of a conscience clause in the 1967 Abortion Act (Fleming et al. 2024). And while the RCM and the Royal College of Obstetricians and Gynaecologists supported decriminalisation legislation in recent debates in the House of Commons, it wasn’t until December 2024 that the RCN spoke out in support of decriminalising abortion (Royal College of Nursing 2024; Royal College of Obstetricians and Gynaecology et al. 2025; Adam 2025).

The Dobbs Court decision in 2022 in U.S.A. (Dobbs 2021 State Health Officer of the Mississippi Department of Health, et al. v. Jackson Women's Health Organisation) is perhaps the most (in)famous example of a right wing, conservative, religious government withdrawing women's rights and access to abortion (Brysk 2025). The Dobbs ruling held that the American Constitution does not provide a right to abortion, and it returned the authority to regulate abortion to the people and their elected representatives. Depending on state laws and regulations, nurses may provide education, counselling and offer general information about abortion procedures and postcare instructions as defined by the care provider. The American Nurses Association (ANA) stated that nurses may discuss abortion as a reproductive health alternative if no other law in that state prohibits the practice (ANA 2022). Nurses are required to discuss relevant sexual and reproductive health choices in an unbiased manner with their patients and support the decisions of their patients regarding sexual health and reproductive choices (ANA 2022). The Dobbs ruling has thrown under graduate and post graduate nurse education into disarray as curricula fall under state law rather than federal law; for example, each nurse and nurse educator has to judge how their individual practice conforms to local law and seek, if they wish, to challenge such (restrictive) laws (Cleveland and Russell 2024).

## 4 Discussion

There are innumerable examples of woman‐centred care in nursing that are influenced by feminism in their goals to empower women both in nursing and as patients (Gagliardi et al. 2019). These models of care build on equal rights for women that were accessed in the mid‐20th century. As Webb (1986) describes what a feminist practice of nursing might encompass (Webb 1986):

• Focus on the context of women's experiences.

• Recognition for the need for women to have greater control over their bodies, health and lives.

• Acknowledgment that sharing with other women the realities of their health experiences would be empowering for both women patients and nurses.

However, the Dobbs v. Jackson Women's Health Organisation ruling in the U.S. and the withdrawal of abortion access in other countries globally (Brysk 2025) have had implications for how nurses care for women. In the U.K., the care Packer experienced did not reflect Webb's aspirations for feminist practice; the care she receives did not appear to be contextualised within her life circumstances. While she took control (by not communicating the truth of her situation), the nurses did not appear to have offered her control against the surveillance she felt herself under—in other words, nurses did not act as advocates in the patriarchal system. The Packer case raises implications for the nurse/midwife‐women relationship because any care that is provided has been understood by individual nurses in this case to be under surveillance. As Lord 2025 argues, ‘The main reason so many individuals and organisations acted with suspicion toward Packer—and in other similar cases—is that the law itself directs and encourages such responses’. At a societal level, there doesn’t appear to be consideration of the issues raised by the increased demand during the pandemic and afterwards for mifepristone/misoprostol, or for the implications of the prosecutions on women's access to abortion in the U.K. This case raises important questions for nurses and midwives, educators and managers to consider.

From the literature reviewed for this article, it is clear that some nurses have coherent, ethical and reasoned responses to the ‘global backlash against access to abortion’ (Malka 2007), although these are limited in number. This is shown in the ways in which the role of nurse practitioner or advanced nurse practitioner has developed in several countries (Canada, U.K.) following the legalisation of medication abortion for first‐trimester pregnancy. While, for nurse practitioners working in abortion care in other countries (U.S.A., Poland), approving medication and assisting with surgical abortions has been challenging for some years and will continue to be so given recent legislation (Brysk 2025). Given these legal restrictions on nurse‐led professional, women‐centred practice and social justice, nurses can find it a challenge to deliver feminist informed care to women seeking abortion in healthcare services which are structured not only by patriarchy, but by capitalism, social class, ethnicity and material conditions (economic conditions, division of labour, education, social inequalities) (Melchior 2004; Melchior 2004).

The structural conditions in which nurses work do not make challenging patriarchy easy for nurses as I argue in this paper. The description of nurses as passive and thus powerless (Traynor 2017) seems apposite given the limited writing which draws on feminist theory and applies to nursing practice in this area. Feminism is inherently political because it is ‘directed at changing existing power relations between women and men in society’ (Weedon 1997, 1). Challenging relationships between not only men and women but also changing the gendered positioning of nursing in relation to medicine, and within the wider healthcare system, would be a risk to existing relationships and ways of working which may be daunting for many nurses. Even where the number of women in medicine (in the U.K. at least) are greater than men, this doesn’t mean that medicine is no longer the instrument of patriarchy. There are some encouraging signs that challenge is possible. Some important papers discussed here have broadly aligned with a feminist praxis encompassing a social equity model of health and nursing care. Yet, as (Simon, 86–87) argues, for nurses to embrace feminist theory would risk them giving up the ‘knowledges and commitments [that] constitute important resources for coping with everyday life’. The political imperative of feminism perhaps explains the paucity of literature on abortion by nurses?

A further challenge for embedding feminist theory in nursing which would inform a more prominent response to the global backlash against abortion, is nursing's relationship with theory. On this relationship, I have written elsewhere about nurses’ reification of the theory‐practice gap which prevents nurses from theorising in practice and practising theoretically. It is worth noting Sarter's argument here about nurses’ relationship with nursing theory (Sarter 2004). Sarter, citing Rutty (1998), argues that theory in nursing has taken such an abstract form that it is not accessible from within the set of discourses available to practising nurses. She suggests that nurses function in a concrete world of bodies; for them, making the leap to disembodied abstractions as in many theories of nursing must seem difficult, if not impossible and certainly without any perceivable usefulness. As a consequence, there appears to be no role for theory in the nurse's lived daily experience. I would extend this to feminist theory and its concerns with praxis and social justice in a working environment where nurses experience daily oppression.

Even if nurses were attracted to feminist theories, it is questionable how successfully individual nurses might deliver feminist informed care in healthcare systems which are patriarchal and structured increasingly through new public management and neoliberalism globally (Orgad and Rottenberg 2023).

In the U.K., in the government's women's health strategy (2022), women's health hubs were commissioned to streamline women's access to sexual and reproductive healthcare for issues such as contraception, menopause and periods. They could have been an opportunity to address historical underfunding in women's healthcare and research. But these hubs have not reduced systemic inequalities in women's health, largely because they fail to address structural oppression from patriarchy, racism, white privilege and other sources of social injustice (Johnstone 2025; All‐Party Parliamentary Group (APPG) on Sexual and Reproductive Health 2025). The APPG argue that women's health has deteriorated due to the impact of funding cuts on services in local communities over the last 20 years and the fragility of the sexual and reproductive health workforce. This workforce is both insufficient and inadequately trained to provide woman‐centred health care tailored to women in their local communities. The APPG point out that many women are currently not able to access the basic sexual and reproductive health services they need, with almost half of British women experiencing poor sexual and reproductive health. In addition, disparities exist between different groups of women based on ethnicity and social class. The lack of access to and provision of sexual and reproductive health care, including abortion services, is described as a post‐code lottery by Taylor et al. (2024). This picture is projected to deteriorate following the dropping of women's health targets from the recently published NHS Plan for England and Wales (Johnstone 2025).

One cannot discuss women's sexual and reproductive health needs, and in particular abortion, without reference to stigma. It is not only structural systems which have to change but the stigmatised nature of sexual and reproductive health (men's as well as women's—but that's another paper) (LeBold and MacDonnell 2020). Access to abortion will not improve until nurses address the shame and stigma that are inherent to women's health experiences (including abortion, Maxwell et al. 2021). Shame and stigma are a recognised part of girls’ and women's health experiences—shame about menstruation, eating disorders, gender‐based violence and reproductive health conditions, such as miscarriage and infertility, as well as mental health (and importantly, perinatal mental health) (Mainey et al. 2020). As Maxwell et al. 2021 argue, nurses need to start presenting abortion as everyday routine health need.

## 5 Conclusions

The relationship between nursing and feminism in its various forms continues to be an ambivalent one as the scholarship and praxis in the nursing literature show in relation to attacks on women's rights and access to, as well as the provision of abortion services. These attacks and restrictions have increased globally over the last 10 years (Brysk 2025); the Dobbs v Jackson ruling in the United States was emblematic of this gradual erosion of women's rights to safe abortion and control over their reproductive health.

Despite the 40 years that have elapsed since Webb described her aspirations for feminist nursing, it is worth noting that Webb argued that it is possible to practice ‘real caring instead of alienated and subservient forms of patient care’ (1986, 15); these latter and dominant forms of care, which reinforce the oppression of women and nurses, are remarkably persistent. The literature discussed in this paper raises questions over how far individual nurses can deliver adequate care in patriarchal healthcare systems. This is unsurprising and may explain why there is no one overall response from nursing to attacks on abortion. While it is significant that attitudes toward nurse‐led care seem to show health care professionals' attitudes toward relaxing medical control over access to abortion are increasingly liberal (Wellings et al. 2024), the recent legal cases referred to in the United Kingdom raise some troubling questions for nurses when caring for women who use abortion self‐medication. The U.K. government's responses (both Conservative and Labour governments) suggest an ambivalence toward actually making abortion easier for women by giving women access to early abortion; neither government has made reform of the Abortion Act a priority.

Looking forward, perhaps we can say that while the relationship between nursing and feminism in its various forms remains ambivalent, the literature discussed here shows that feminism could be foundational for ethical and emancipatory nursing practice in women's health and abortion services. An ethical and emancipatory model for nursing would foreground all women's experiences, recognise the need for women to have control over their bodies, health and lives and address white privilege. A feminist praxis thus offers a guide for woman‐centred nursing over the next 50 years if introduced consistently in professional preparation programmes and post‐registration education courses.

## Author Contributions

**Helen T. Allan:** conceptualisation, writing – original draft, writing – review and editing.

## Funding

The author has nothing to report.

## Ethics Statement

Not relevant as this is not original research.

## Conflicts of Interest

The author declares no conflicts of interest.

## Data Availability

Data sharing not applicable to this article as no datasets were generated or analysed during the current study.
